# Efficacy Evaluation of a Bivalent Vaccine Containing Porcine Circovirus Type 2b and *Mycoplasma hyopneumoniae* Against an Experimental Dual Challenge

**DOI:** 10.3389/fvets.2021.652313

**Published:** 2021-04-30

**Authors:** Yongjun Ahn, Siyeon Yang, Taehwan Oh, Kee Hwan Park, Hyejean Cho, Jeongmin Suh, Chanhee Chae

**Affiliations:** Department of Veterinary Pathology, College of Veterinary Medicine, Seoul National University, Seoul, South Korea

**Keywords:** *mycoplasma hyopneumoniae*, porcine circovirus type 2, porcine respiratory disease complex, bivalent vaccine, experimental challenge

## Abstract

The purpose of this study was to evaluate the efficacy of a new, single-dose bivalent vaccine containing porcine circovirus type 2b (PCV2b) and *Mycoplasma hyopneumoniae* against a dual PCV2b and *M. hyopneumoniae* challenge. At −25 days post challenge (dpc, 10 days of age), one pig group (designated as the vaccinated/challenged group) received a single, 1.0 ml dose of bivalent vaccine. Pigs in both the vaccinated/challenged and unvaccinated/challenged groups were then inoculated intranasally with PCV2b and *M. hyopneumoniae* at 0 dpc (35 days of age). Pigs in vaccinated/challenged group induced significantly higher levels of neutralizing antibodies against PCV2b and cell-mediated immunity against PCV2b and *M. hyopneumonia* when compared with pigs in unvaccinated/challenged group. The vaccination of pigs with a bivalent vaccine also reduced PCV2b viremia, reduced mycoplasmal nasal shedding, and decreased the severity of both lung and lymphoid lesions for PCV2b and *M. hyopneumoniae* infection, respectively. The results of this study demonstrated that the evaluated bivalent vaccine was effective in protecting pigs against PCV2b and *M. hyopneumoniae* infection.

## Introduction

Pneumonia caused by multiple infectious agents has been described with the term “Porcine Respiratory Disease Complex (PRDC).” PRDC infection is categorized by reduced pig performance, increased medication costs to the producer, and an increased mortality rate during the finishing process (15 to 20 weeks of age) (1). The etiology of PRDC is extremely diverse and occurs in both all-in-all-out as well as in continuous production systems. The main three causative agents of PRDC are porcine circovirus type 2 (PCV2), porcine reproductive and respiratory syndrome virus (PRRSV), and *Mycoplasma hyopneumoniae*, which are known to be responsible for serious economic damages within the global pig industry (1). Among the three, co-infection of pigs with PCV2 and *M. hyopneumoniae* is the most frequent combination in field PRDC cases and is the most rapidly increasing within the Asian pork industry (2). Vaccination is routinely implemented to control PRDC in relation to PCV2 and *M. hyopneumoniae* infection (3). This increase in vaccination numbers has led to a demand for single-dose bivalent vaccines containing PCV2 and *M. hyopneumoniae*. This experimental challenge study was designed to help meet this demand by evaluating the efficacy of a new bivalent PCV2b and *M. hyopneumoniae* vaccine (Circo/MycoGard, Pharmgate Animal Health, Wilmington, NC, USA) containing killed Baculovirus vector and *M. hyopneumoniae* bacterin vaccine with a trivalent-adjuvanted formulation against an experimental challenge of PCV2b and *M. hyopneumoniae*.

## Materials and Methods

A total of 24 colostrum-fed, cross-bred, conventional piglets were purchased at 7 days of age from a PRRSV- and *M. hyopneumoniae*-free commercial farm. The negative status of the farm was based on serological testing of the breeding herd, and long term clinical and slaughter history. Sows residing on the commercial farm were naïve to vaccination against PCV2 and *M. hyopneumoniae*, while all piglets were vaccinated against both pathogens at 21 days of age. The pigs were weaned early at 7 days of age before vaccination of PCV2 and *M. hyopneumoniae* and selected for this study based on their seronegative results for PRRSV (IDEXX PRRS X3 Ab test, IDEXX Laboratories Inc., Westbrook, Maine, USA), *M. hyopneumoniae* (*M*. *hyo*. Ab test, IDEXX Laboratories Inc.), and PCV2 (PCV2 Ab Mono Blocking, Synbiotics, Lyon, France). In addition, negative results for viral and mycoplasmal infections were also obtained for PCV2 and PRRSV from sera samples and for *M. hyopneumoniae* from nasal swabs as tested by real-time polymerase chain reaction (PCR) (4–6).

A total of 24 pigs were randomly allocated into three groups that contained eight piglets per groups (4 = male and 4 = female). Three rooms, uniform in design that allowed free access to feed and water troughs each contained ten pens in facility of Seoul National University. Four pigs were randomly assigned to the pens from each of the three groups. All randomizations were performed using the random number generator function (Excel, Microsoft Corporation, Redmond, WA, USA).

At −25 days post challenge (dpc, 10 days of age), pigs in the Vaccinated/Challenged (Vac/Ch) group were vaccinated intramuscularly on the right side of the neck with 1.0 ml of a bivalent vaccine containing PCV2b and *M. hyopneumoniae* (Circo/MycoGard, Serial No: CMG-18007, Expiration date: 02.28.2020). Pigs in the Unvaccinated/Challenged (UnVac/Ch) and Unvaccinated/Unchallenged (UnVac/UnCh) groups received a 1.0 ml injection of phosphate buffered saline (PBS, 0.01 M, pH 7.4) in the same anatomical location as the Vac/Ch group.

At 0 dpc (35 days old), pigs in the Vac/Ch and UnVac/Ch groups were challenged by inoculation with PCV2b (strain SNUVR000463, GenBank no. KF871068). Five hours later, an *M. hyopneumoniae* (strain SNU98703) challenge was administered. Co-infection with PCV2b (strain SNUVR000463) and *M. hyopneumoniae* (strain SNU98703) induced severe pneumonia in lungs and lymphoid depletion in the lymph node in infected pigs (7). The wait interval was performed to avoid the mixture of two pathogens which could have resulted in an infectivity decrease. During the PCV2b challenge, a 3 ml inoculation containing 1.2 × 10^5^ (50% tissue culture infective dose (TCID_50_)/ml) was administered intranasally. Five hours post-PCV2 inoculation, pigs were intramuscularly anesthetized with a mixture of 2.2 mg/kg xylazine hydrochloride (Rompun, Bayer), 2.2 mg/kg tiletamine hydrochloride, and 2.2 mg/kg zolazepam hydrochloride (Zoletil 50, Virbac). Then, pigs were inoculated intratracheally with 7 ml of *M. hyopneumoniae* (strain SNU98703) culture medium containing 10^7^ color changing units (CCU)/ml. All study methods were approved previously by the Seoul National University Institutional Animal Care and Use, and Ethics Committee (SNU-181018-8-2).

At 21 dpc (56 day old), all pigs were sedated with an intravenous injection of sodium pentobarbital prior to euthanasia by electrocution as previously described (8). Tissues were collected from each pig at necropsy. Tissue preparation included fixation in a 10% neutral buffered formalin solution for 24 h before they were routinely processed and embedded in paraffin.

Pigs were monitored daily and scored weekly for clinical signs as previously described (9). Scores ranged from 0 to 6: 0 = normal; 1 = rough haircoat; 2 = rough haircoat and dyspnea; 3 = mild dyspnea and abdominal breathing; 4 = moderate dyspnea and abdominal breathing; 5 = severe dyspnea and abdominal breathing; 6 = death.

Pigs were weighed at 10 (−25 dpc) and 56 (21 dpc) days of age. Average daily gain (ADG) was calculated as the difference between the starting and final weight divided by the number of days spanning the duration of the stage, and included data for pigs that died or were removed from the study.

Blood and nasal swabs were collected from all pigs at −25, −14, 0, 7, 14, and 21 dpc. A commercial kit (QIAamp DNA Mini Kit, QIAGEN, Valencia, CA, USA) was use to extract DNA from serum samples and nasal swabs. The number of genomic DNA copies for *M. hyopneumoniae* was quantified by real-time PCR (4). To construct a standard curve, real-time PCR was performed in quadruplicate in 10-fold serial dilution of chromosomal DNA from *M. hyopneumoniaes* strain SNU98703, with concentrations ranging from 10 ng/μl to 1 fg/μl. One fetogram of chromosomal DNA from *M. hyopneumoniae* is considered to be ~one genome equivalent (10). A negative control was included in each run using double distilled water as the template.

The number of genomic DNA copies for PCV2b was quantified by real-time PCR (3). To construct a standard curve, real-time PCR was performed in quadruplicate in two different assays: (i) 10-fold serial dilutions of the PCV2b plasmid were used as the standard, with concentrations ranging from 10^10^ to 10^2^ copies/ml, and (ii) 10-fold serial dilutions of PCV2b cultured in PCV1-free PK-15 cells were used at concentrations ranging from 10^4.5^ TCID_50_/ml to 10^−3.5^ TCID_50_/ml. The PCV2b plasmid was prepared as described previously (3). Culture supernatants of PCV1-free PK-15 cells were used as negative control.

The presence of *M. hyopneumoniae* and PCV2 antibodies were evaluated in serum samples by use of commercially available enzyme-linked immunosorbent assay (ELISA) kits (*M. hyo* Ab test, IDEXX Laboratories Inc and SERELISA PCV2 Ab Mono Blocking, Synbiotics). Testing was conducted in accordance with each manufacturer's kit instructions, where samples were considered as positive for *M. hyopneumoniae* antibody if the sample-to-positive (S/P) ratio was ≥ 0.4 and as positive for PCV2 antibodies if the reciprocal ELISA titer was > 350. Serum samples were also tested for serum virus neutralization against PCV2b (11).

Inactivated *M. hyopneumoniae* and PCV2b antigens using challenge strains for *M. hyopneumoniae* and PCV2b were prepared for enzyme-linked immunospot (ELISpot) assay as previously described (12, 13). An ELISpot assay was conducted to measure the numbers of *M. hyopneumoniae*- and PCV2b-specific interferon-γ secreting cells (IFN-γ-SC). Peripheral blood mononuclear cells (PBMC) were stimulated with inactivated *M. hyopneumoniae* and PCV2b antigens and results reported as the number of IFN-γ-SC per million PBMC (12, 14). Phytohemagglutinin (Roche Diagnostics GmbH, Mannheim, Germany) and PBS used as a positive and negative control, respectively.

Lung lesion scoring was performed for *M. hyopneumoniae* infection and lymphoid lesion for PCV2b infection by two veterinary pathologists. Severity of lung lesion was scored (0 to 6) based on peribronchiolar and perivascular lymphoid tissue hyperplasia (15). Severity of lymphoid lesion was scored (0 to 5) based on lymphoid depletion and granulomatous inflammation (16).

All real-time PCR data was transformed to log_10_ values prior to statistical analysis. The Shapiro-Wilk test evaluated data for normal distribution. One-way analysis of variance (ANOVA) was used to examine whether there were statistically significant differences at each time point within the three groups. If a one-way ANOVA test resulted in a statistical significance, data was further evaluated by conducting a *post-hoc* test for a pairwise comparison with Tukey's adjustment. The Kruskal-Wallis test was performed if the normality assumption was not met. Kruskal Wallis test results which showed a statistical significance were further evaluated with the Mann-Whitney test to include Tukey's adjustment to compare the differences among the groups. The Pearson's correlation coefficient was used to assess the correlation of PCV2b viremia with neutralizing antibody titers against PCV2b, PCV2b viremia with PCV2b-specific IFN-γ-SC, and nasal shedding of *M. hyopneumoniae* and *M. hyopneumoniae*-specific IFN-γ-SC. Results were reported in *P*-value where a value of *P* < 0.05 was considered to be significant.

## Results

The mean scores for respiratory disease were significantly lower (*P* < 0.05) in pigs from the Vac/Ch group (mean ± standard deviation, 0.38 ± 0.52 for 7 dpc, 0.75 ± 0.71 for 14 dpc, and 0.63 ± 0.52 for 21 dpc) when compared with the UnVac/Ch group (mean ± standard deviation, 1.00 ± 0.53 for 7 dpc, 2.13 ± 0.83 for 14 dpc, and 2.13 ± 0.83 for 21 dpc) at 7, 14, and 21 dpc. Pigs from the Group 3 remained normal throughout the experiment.

The body weight of the pigs did not differ significantly among three groups at study day 0 (the time of vaccination, 10 days of age). Pigs from the Vac/Ch (mean ± standard deviation, 301.1 ± 11.3) and UnVac/UnCh (mean ± standard deviation, 309.2 ± 4.8) groups had significantly higher (*P* < 0.05) ADG (unit = gram/pig/day) between 10 and 56 days of age when compared with those from UnVac/Ch (mean ± standard deviation, 277.5 ± 16.4) group.

**Figure 1:**
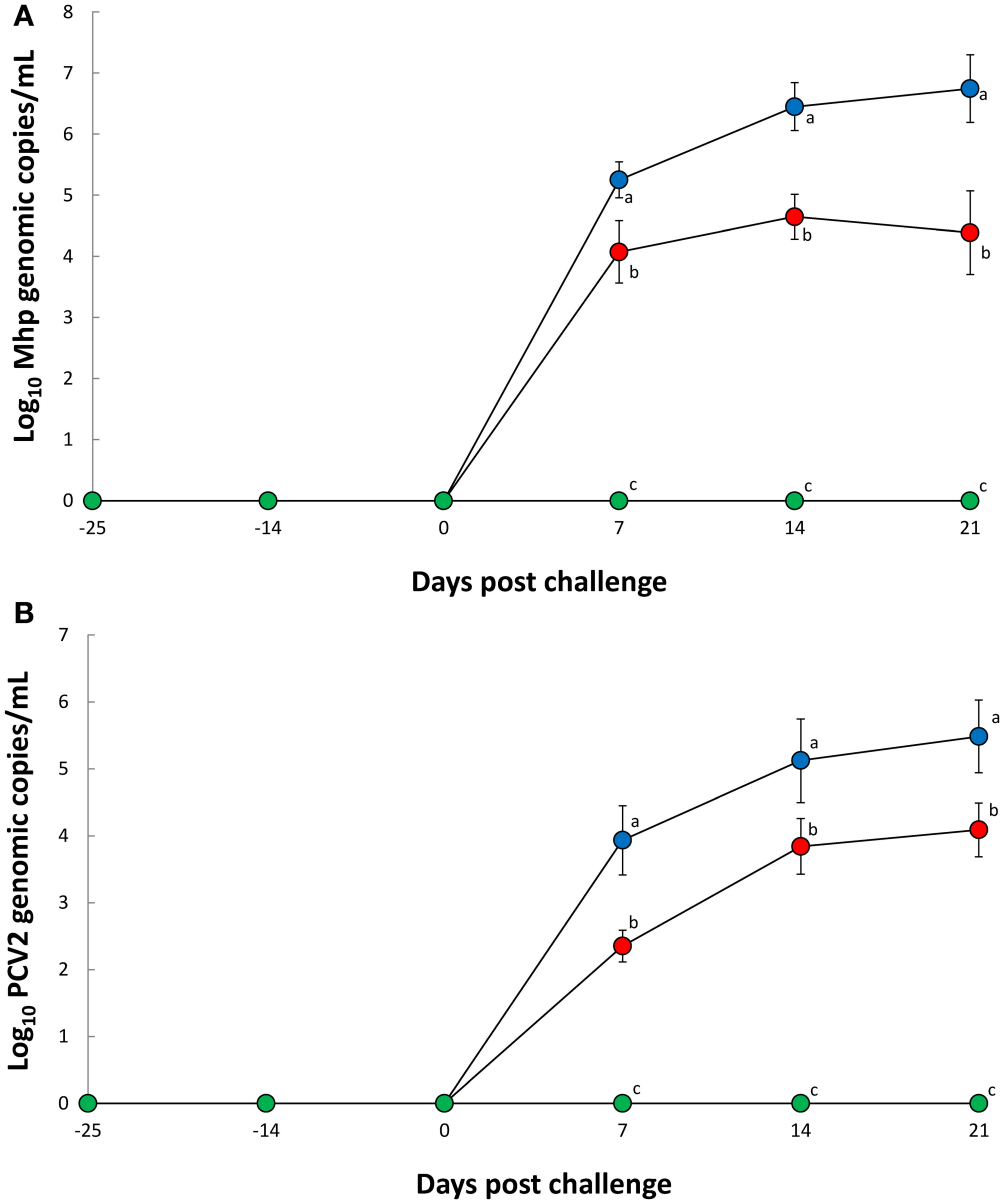
Mean values of the genomic copy number of *Mycoplasma hyopneumoniae* DNA in nasal swabs **(A)** and porcine circovirus type 2b in blood **(B)** from Vac/Ch (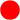), UnVac/Ch (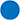), and UnVac/UnCh (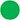) groups. Variation is expressed as the standard deviation. Different superscripts (a, b, and c) indicate significant (*P* < 0.05) difference among three groups.

Nasal swabs evaluation between 7 and 21 dpc reported significantly less (*P* < 0.05) *M. hyopneumoniae* genomic copies in pigs from the Vac/Ch group when compared with the UnVac/Ch group (Figure 1A). Blood sample evaluation from the same timeframe (7 to 21 dpc) also reported significantly less (*P* < 0.05) PCV2d genomic copies in the Vac/Ch group when compared with the UnVac/Ch group (Figure 1B). No *M. hyopneumoniae* or PCV2 were detected in the pigs from the UnVac/UnCh group.

**Figure 2:**
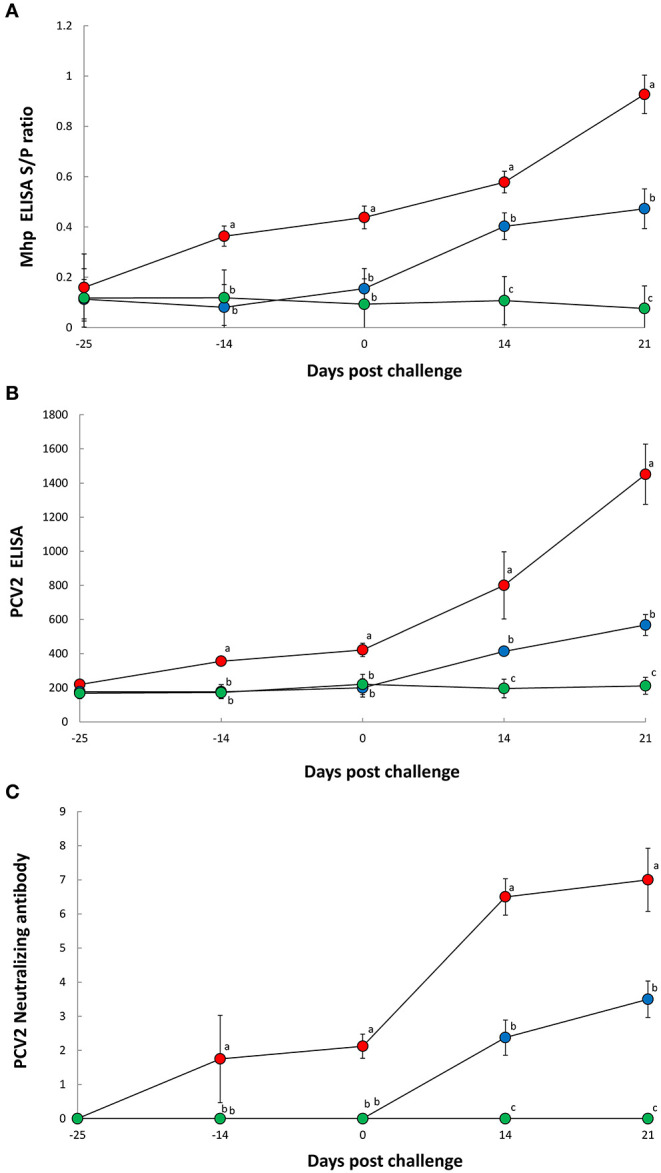
ELISA antibody levels of *Mycoplasma hyopneumoniae* **(A)**, ELISA antibody levels of porcine circovirus type 2 **(B)**, and neutralizing antibody titers against porcine circovirus type 2b **(C)** in serum from Vac/Ch (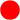), UnVac/Ch (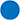), and UnVac/UnCh (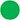) groups. Variation is expressed as the standard deviation. Different superscripts (a, b, and c) indicate significant (*P* < 0.05) difference among three groups.

Pigs in the Vac/Ch group had a significantly higher (*P* < 0.05) *M. hyopneumoniae* ELISA S/P ratio in their −14 to 21 dpc serum samples when compared with the UnVac/Ch group (Figure 2A). Pigs in the Vac/Ch group had a significantly higher (*P* < 0.05) PCV2 ELISA titer from −14, to 21 dpc in their serum samples when compared with the UnVac/Ch group (Figure 2B). Pigs in the Vac/Ch group had significantly higher (*P* < 0.05) neutralizing antibody titers against PCV2b from −14 to 21 dpc when compared with the UnVac/Ch group (Figure 2C). There was a correlation between number of genomic copies of PCV2b in the blood and neutralizing antibody titers against PCV2b (*r* = −0.810, *P* = 0.015). *M. hyopneumoniae* and PCV2 antibodies were not detected in pigs from the UnVac/UnCh group.

**Figure 3:**
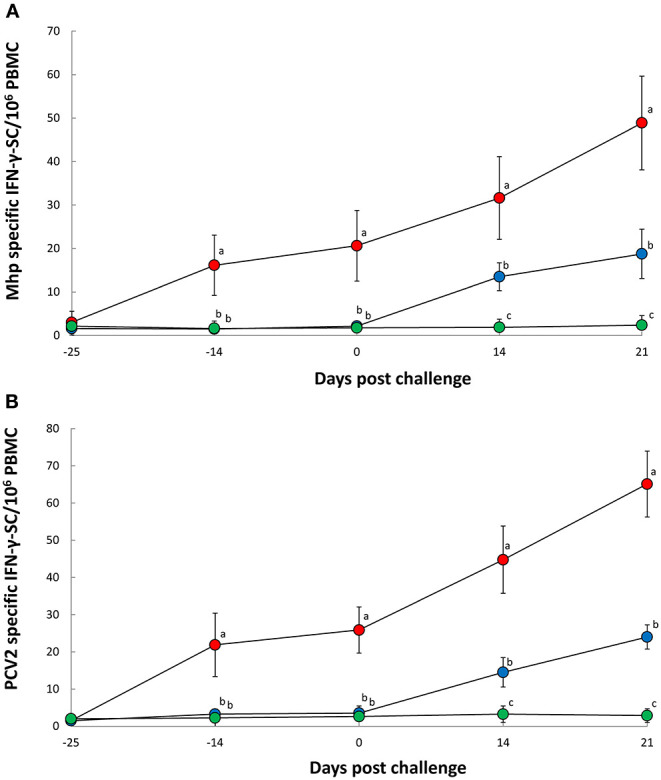
Frequency of *Mycoplasma hyopneumoniae*-specific interferon-γ secreting cells (IFN-γ-SC) **(A)** and porcine circovirus type 2b-specific IFN-γ-SC **(B)** in peripheral blood mononuclear cells (PBMC) from Vac/Ch (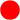), UnVac/Ch (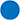), and UnVac/UnCh (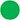) groups. Variation is expressed as the standard deviation. Different superscripts (a, b, and c) indicate significant (*P* < 0.05) difference among three groups.

Pigs in the Vac/Ch group had a significantly higher (*P* < 0.05) number of *M. hyopneumoniae*- (Figure 3A) and PCV2b- (Figure 3B) specific IFN-γ-SC in their PBMC from −14, 0, 14, and 21 dpc when compared with the UnVac/Ch group. There was a correlation between the number of genomic copies of *M. hyopneumoniae* in the nasal swabs and numbers of *M. hyopneumoniae*-specific IFN-γ-SC (*r* = −0.758, *P* = 0.029). *M. hyopneumoniae* and PCV2b-specific IFN-γ-SC were not detected in pigs from the UnVac/UnCh group.

Pigs in the Vac/Ch group had significantly lower (*P* < 0.05) macroscopic (mean ± standard deviation, 17.4 ± 5.3) and microscopic (mean ± standard deviation, 0.9 ± 0.34) lung lesion scores for *M. hyopneumoniae* infection at 21 dpc when compared with the UnVac/Ch group with macroscopic (mean ± standard deviation, 45.8 ± 3.2) and microscopic (mean ± standard deviation, 3.5 ± 0.4) lung lesion scores. Pigs in the Vac/Ch group also had significantly lower (*P* < 0.05) microscopic lymphoid lesions scores (mean ± standard deviation, 0.7 ± 0.2) for PCV2b infection at 21 dpc when compared with the UnVac/Ch group with microscopic lymphoid lesions scores (mean ± standard deviation, 3.5 ± 0.5). Macroscopic and microscopic lung lesions, and microscopic lymphoid lesions were not observed in pigs from the UnVac/UnCh group.

## Discussion

The study results demonstrated that the evaluated bivalent vaccine containing PCV2b and *M. hyopneumoniae* against a dual challenge of PCV2b and *M. hyopneumoniae* is efficacious in protecting pigs. The evaluation of growth performance was identified as the critical factor in determining the efficacy of this bivalent vaccine as PCV2 and *M. hyopneumoniae* co-infection is mainly characterized by poor growth performance. Vaccination of pigs with the evaluated bivalent vaccine resulted in improved growth performance when compared with unvaccinated pigs.

The bivalent vaccine tested in this study was administered to piglets at 10 days of age. There was potential, therefore, for interference with maternally derived antibodies. In this study, interference by maternally derived antibodies was not evaluated as pigs tested as seronegative against PCV2 prior to study initiation. It can be deduced, however, maternally derived antibodies did not significantly interfere with the induction of both humoral and cell-mediated immunity in piglets post PCV2 vaccination (17).

*M. hyopneumoniae* is known to have a potentiating effect on the level of PCV2 viremia in pigs co-infected with *M. hyopneumoniae* and PCV2 (15). The reduction of PCV2b viremia was therefore an effect of bivalent vaccination but not contribute to an immunosuppressive effect of *M. hyopneumoniae* infection in vaccinated pigs challenged with PCV2b and *M. hyopneumoniae*.

Neutralizing antibodies and IFN-γ-SC are responsible for the reduction of PCV2 viremia (18–20), where these levels are considered measurements of protective immunity (19, 20). The amount of neutralizing antibodies present correlated with the PCV2b viremia reduction in the present study, while frequency of IFN-γ-SC did not. On other hands, it should be noted that the protective immunity mechanism against *M. hyopneumoniae* is not fully understood, and therefore caution should always be exercised with evaluating it against study conclusions. Cell-mediated immunity has proven to play an important role in controlling *M. hyopneumoniae* infection (21). In the present study, cell-mediated immunity as measured by IFN-γ-SC correlated with a significant reduction in the amount of *M. hyopneumoniae* loads through nasal shedding. This study demonstrated that pig vaccination and challenge induced high levels of protective immunity, reduced the amount of PCV2b viremia and severity of lymphoid lesions, and reduced the amount of *M. hyopneumoniae* nasal shedding and severity of lung lesions.

## Data Availability Statement

The original contributions generated for the study are included in the article/supplementary material, further inquiries can be directed to the corresponding authors.

## Ethics Statement

The animal study was reviewed and approved by Seoul National University Institutional Animal Care and Use Committee (SNU-181018-8-2).

## Author Contributions

YA performance of the experimental trials and data analysis and writing of the manuscript. SY, TO, and KP preparation of the inoculum and lab analysis. HC immune analysis. JS pathological analysis. CC development of protocol, design of the study, review of the final manuscript, and approval for publication. All authors read and approved the final manuscript.

## Conflict of Interest

The authors declare that the research was conducted in the absence of any commercial or financial relationships that could be construed as a potential conflict of interest.
